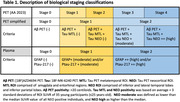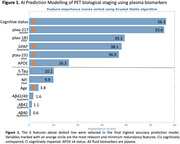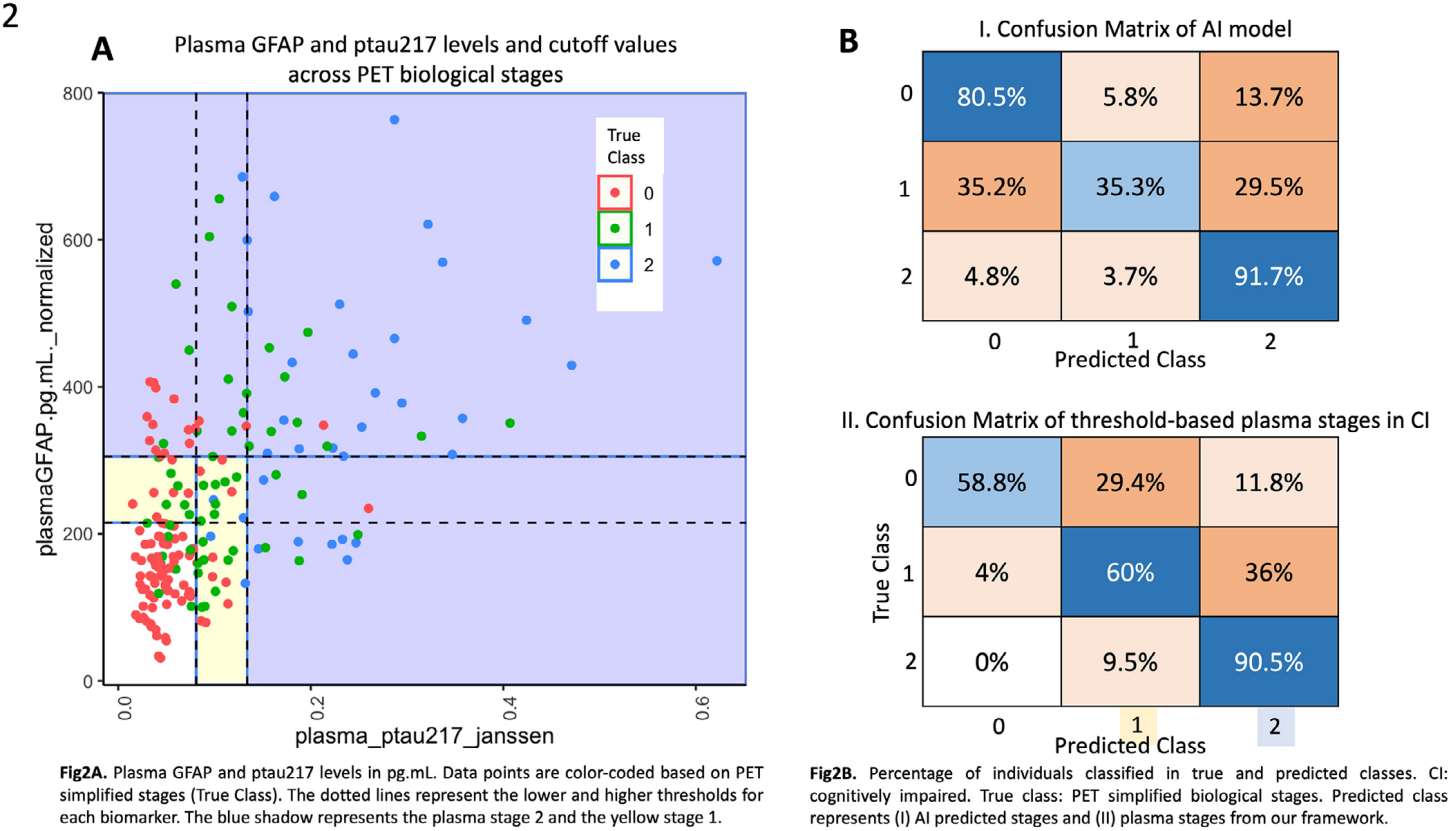# Performance of an AI‐based prediction model versus stratified thresholds using plasma biomarkers for biological staging of AD

**DOI:** 10.1002/alz.092879

**Published:** 2025-01-09

**Authors:** Marina Scop Madeiros, Pamela C.L. Ferreira, Guilherme Bauer‐Negrini, Guilherme Povala, Bruna Bellaver, Cristiano Schaffer Aguzzoli, Carolina Soares, Hussein Zalzale, Markley Oliveira, Matheus Scarpatto Rodrigues, Sarah Abbas, Livia Amaral, Cynthia Felix, Pampa Saha, Emma Patrice Ruppert, Devin J Fine, Juli Cehula, Madeleine Bloomquist, Firoza Z Lussier, Joseph Therriault, Cécile Tissot, Andrea L. Benedet, Nesrine Rahmouni, Dana Tudorascu, Pedro Rosa‐Neto, Thomas K Karikari, Lucas Porcello Schilling, Tharick A. Pascoal

**Affiliations:** ^1^ University of Pittsburgh, Pittsburgh, PA USA; ^2^ Pontifical Catholic University of Rio Grande do Sul, Porto Alegre, RS Brazil; ^3^ Department of Psychiatry, University of Pittsburgh School of Medicine, Pittsburgh, PA USA; ^4^ Brain Institute of Rio Grande do Sul, PUCRS, Porto Alegre, RS Brazil; ^5^ Global Brain Health Institute, San Francisco, CA USA; ^6^ Translational Neuroimaging Laboratory, The McGill University Research Centre for Studies in Aging, Montréal, QC Canada; ^7^ McGill University, Montreal, QC Canada; ^8^ McGill University Research Centre for Studies in Aging, Montreal, QC Canada

## Abstract

**Background:**

The potential clinical utility of plasma biomarkers for biological staging of AD demands definition and validation of cutoff values. Plasma ptau‐217 and GFAP have accurately predicted core pathological changes such as tau aggregation and amyloid (Aβ) deposition, being proposed as complementary biomarkers. Thus, we aim to test a staging framework with plasma GFAP and ptau‐217 using cuttof values to predict Aβ/Tau PET stages and compare its performance with an artificial intelligence (AI) prediction model.

**Methods:**

We included 362 individuals from TRIAD cohort and classified by Aβ and Tau PET in 5 biological stages and in 3 PET simplified stages (Table 1), representing the gold‐standard. For the AI model, we performed feature selection of 12 variables (Figure 1A) and repeated the model removing lower importance variables until reaching highest accuracy. For the classic thresholding method, we selected plasma GFAP(Quanterix) and plasma ptau‐217(Janssen) and defined lower and higher thresholds with ROC curves for PET stages discrimination in cognitively impaired individuals. Plasma stages were then defined for 199 participants that had GFAP and ptau‐217 data (Figure 2A).

**Results:**

Our study revealed that the AI Ensemble Boosted Trees model was the most accurate in distinguishing PET simplified stages, utilizing 6 key variables (Figure 1). Through 5‐fold cross‐validation, the model achieved a validation AUC of 0.91 for predicting stage‐2 and 0.83 for stage‐0, with a consistent test AUC of 0.94 for both stages. Notably, plasma ptau‐217 emerged as the most significant predictor among the ptau‐x variables, closely followed by GFAP (Figure 1). Our analysis using plasma thresholds for GFAP and ptau‐217 (Figure 2A) yielded AUCs of 0.78 for stage‐0 and 0.85 for stage‐2 predictions. A comparative assessment of confusion matrices showed similar accuracies (Figure 2B). Importantly, our threshold‐based classification accurately detected 96% of early/stage‐1 cases as positive and correctly avoided any misclassification of late/stage‐2 cases as stage‐0.

**Conclusions:**

Our threshold‐based framework, which utilizes plasma GFAP and ptau‐217, exhibits potential for the biological staging of AD, having achieved comparable accuracy to AI‐based methods that employ multiple plasma biomarkers and demographics to detect PET stages. This framework is straightforward, relies solely on two blood biomarkers and clinically‐stratified thresholds, and holds promise for implementation in clinical practice.